# Effects of matching diet type to obesity-related genotype on body composition changes in women during a six-month resistance-exercise training and walking program

**DOI:** 10.1186/1550-2783-12-S1-P16

**Published:** 2015-09-21

**Authors:** A Coletta, B Sanchez, A O'Connor, R Dalton, S Springer, M Koozehchian, YP Jung, S Simbo, M Cho, C Goodenough, A Reyes, R Sowinski, L Wilkins, C Rasmussen, RB Kreider

**Affiliations:** 1 Exercise & Sport Nutrition Lab, Texas A&M University, College Station, TX, USA; 2 Interleukin Genetics, Waltham, MA, USA

## Background

We recently reported [1] that correctly matching diet type to some obesity-related genes promoted greater fat loss during the first 3 months of a diet and exercise intervention. This study examined whether these changes were observed following a 6-month diet and exercise training program.

## Methods

Fifty sedentary, obese women (41.6 ± 12 yrs, 35.4 ± 8 kg/m^2^) were assigned to diet groups based on five obesity-related genetic variants from four genes prominently associated with obesity (FABP2, PPARG, ADRB2, ADRB3). Participants were either truly matched (T) to their diet group based on genotype (n = 28) or falsely matched (F) based on genotype (n = 22). Prescribed diets consisted of 1,500 kcal/d and included carbohydrate:fat:protein percentages of 30:25:45 (H) or 20:35:45 (L). Participants performed a supervised circuit-style resistance-exercise program four days/week and a walking program consisting of 10,000 steps/day, three days/week. Body weight and duel energy X-ray absorptiometry (DXA) body composition measures were obtained at baseline, 4, 8, 12, 16, 20, and 24 weeks. Data were analyzed by MANOVA, with baseline body weight and body composition values used as covariates to normalize baseline differences between groups. Data are presented as changes from baseline at each time point, respectively.

## Results

MANOVA revealed an overall Wilks' Lamda time effect (p < 0.001) with no significant time by diet (p = 0.51), time × gene type (0.84), or time × diet × gene type (0.81) effects observed. Univariate analysis revealed that the exercise and diet interventions promoted significant reductions in weight (-5.36 ± 5.0 kg, p < 0.001), fat mass (-4.53 ± 3.6 kg, p < 0.001), and body fat (-2.88 ± 2.7 %, p < 0.001) with a trend toward a reduction in fat free mass (-0.65 ± 2.3 kg, p < 0.071). When baseline body weight and DXA body composition variables were used as covariates, Wilks' Lambda time × diet (p = 0.098) tended to differ, a time × gene type interaction was observed (p = 0.011), while no differences were seen in time × diet × gene type (p = 0.18). Univariate analyses revealed some trends in time × diet changes in weight (H -2.03 ± 1.7, -3.13 ± 2.6, -4.17 ± 3.3, -4.62 ± 4.0, -4.75 ± 4.6, -4.41 ± 5.1; L -2.47 ± 1.8, -3.66 ± 2.4, -4.56 ± 3.1, -5.49 ± 3.9, -5.89 ± 4.5, -6.17 ± 4.8 kg, p_q _= 0.02), fat mass (H -1.53 ± 1.4, -2.67 ± 2.3, -3.63 ± 2.5, -3.73 ± 2.8, -4.14 ± 3.6, -3.95 ± 3.6; L -1.31 ± 1.6, -2.66 ± 2.2, -3.22 ± 2.5, -4.32 ± 2.9, -4.60 ± 3.0, -5.03 ± 3.7 kg, p_q _= 0.10), FFM (H -0.49 ± 1.2, -0.58 ± 1.5, -0.52 ± 1.8, -0.68 ± 2.4, -0.51 ± 2.2, -0.31 ± 2.3; L -0.95 ± 1.5, -0.73 ± 1.9, -1.20 ± 1.9, -0.86 ± 2.0, -1.03 ± 2.6, -0.94 ± 2.4 kg, p = 0.14), or body fat (H -0.50 ± 1.9, -1.51 ± 1.8, -2.32 ± 2.2, -2.22 ± 2.0, -2.68 ± 2.5, -2.65 ± 2.3; L -0.46 ± 1.5, -1.55 ± 2.1, -1.67 ± 2.4, -2.56 ± 2.5, -2.78 ± 2.7, -3.08 ± 3.0 %, p_q _= 0.13) generally in favor of the more carbohydrate restricted diet. Some trends were also seen in time × gene type changes in weight (T -2.06 ± 1.8, -2.91 ± 2.6, -3.99 ± 3.3, -4.83 ± 4.0, -5.07 ± 4.6, -5.15 ± 5.1; F -2.54 ± 1.7, -4.05 ± 2.2, -4.88 ± 2.9, -5.43 ± 3.8, -5.73 ± 4.6, -5.62 ± 5.0 kg, p = 0.20), fat mass (T -1.39 ± 1.6, -2.42 ± 2.2, -3.15 ± 2.4, -3.74 ± 2.8, -4.27 ± 3.3, -4.18 ± 3.3; F -1.45 ± 1.3, -2.98 ± 2.1, -3.74 ± 2.6, -4.45 ± 2.9, -4.54 ± 3.3, -4.98 ± 4.0 kg, p_q _= 0.19), FFM (T -0.50 ± 1.6, -0.40 ± 1.5, -0.78 ± 1.8, -0.78 ± 2.0, -0.60 ± 1.9, -0.81 ± 2.3; F -1.04 ± 1.1, -0.99 ± 2.0, -1.02 ± 1.9, -0.76 ± 2.4, -1.04 ± 3.0, -0.45 ± 2.3 kg, p = 0.01), and body fat (T -0.76 ± 1.6, -1.58 ± 2.0, -1.98 ± 2.3, -2.26 ± 2.1, -2.84 ± 2.2, -2.70 ± 2.4; F -0.12 ± 1.8, -1.47 ± 2.0, -1.96 ± 2.3, -2.60 ± 2.5, -2.60 ± 3.1, -3.12 ± 3.0 %, p_q _= 0.05) with those in the false match group observing generally greater changes.

## Conclusion

Results revealed that participants following a more carbohydrate restricted diet experienced significantly greater weight loss and slightly greater body composition changes. Matching diet based on gene-type exhibited better retention of fat free mass, with no significant differences between groups in changes in weight or fat mass. While changes in body fat percentage were similar between groups throughout the intervention, by week 24 individuals in the false match group experienced slightly greater loss.
